# Irvine–Gass Syndrome Personalized Treatment Outcomes: A Retrospective Single-Center Cohort Study

**DOI:** 10.3390/jpm15090428

**Published:** 2025-09-05

**Authors:** Lorenzo Tomaschek, Laura Hoffmann, Robert Katamay, David Stocker, Asan Kochkorov, Katja Hatz

**Affiliations:** 1 Vista Augenklinik Binningen, 4102 Binningen, Switzerland; 2 Faculty of Medicine, University of Basel, 4056 Basel, Switzerland; 3 Vista Augenklinik Zürich, 8001 Zürich, Switzerland; 4 School of Engineering, Zürcher Hochschule für Angewandte Wissenschaften, 8021 Zürich, Switzerland

**Keywords:** Irvine–Gass syndrome, pseudophakic cystoid macular edema, topical therapy, systemic therapy, central subfield thickness, risk factors, personalized treatment

## Abstract

Irvine–Gass syndrome (IGS) is a macular edema that is mostly observed after cataract surgery, also known as pseudophakic cystoid macular edema (PCME). To date, there are still no standardized guidelines for its treatment. **Background/Objectives**: This study aimed to compare the efficacy of local and systemic treatments on the resolution of Irvine–Gass Syndrome as well as the therapeutic outcomes of patients with known risk factors such as diabetes and arterial hypertension in order to be able to personalize treatment regimens for each patient. **Methods**: A total of 136 eyes were followed for a mean of 9.7 ± 15.2 months, with patients divided as follows: those who received only local treatment (LT), those who received systemic treatment (ST), those with cardiovascular diseases (CV), and those without cardiovascular diseases (NCV). We compared the time from the diagnosis of IGS to fully recovered edema (no sub- or intraretinal fluid), central subfield thickness (CST, as evaluated using optical coherence tomography), visual acuity (VA), and intraocular pressure (IOD) in each group. The time from diagnosis to resolution was measured from the initiation of therapy to the full resolution of edema. **Results**: A total of 136 eyes were examined. The mean CST significantly decreased in the LT (*n* = 75) (458.3 ± 96.5 µm to 320 ± 39.5 µm (*p* < 0.01)) and ST (*n* = 61) groups (519.3 ± 121.6 µm to 337.2 ± 70.6 µm (*p* < 0.01)) from baseline to 12 months, with no significant difference (*p* = 0.92). The mean VA significantly increased in both groups from baseline to 12 months (LT: 69.1 ± 11.9 to 80.4 ± 6.6 letters (*p* < 0.01); ST: 65.1 ± 11.8 to 78.5 ± 6.8 letters (*p* < 0.01)). The mean time to the resolution of edema was significantly shorter in the LT group (*p* < 0.05). There were no significant differences in the CST decrease, VA gain, or time to edema resolution between the CV and NCV patients. **Conclusions**: In regard to the non-inferiority of local treatment, a personalized approach for each patient should be considered, and systemic treatment must be critically evaluated to determine possible side effects.

## 1. Introduction

The most common postoperative complication of cataract surgery is still Irvine–Gass syndrome (IGS), also known as pseudophakic cystoid macular edema (PCME), which causes severe vision impairment. In 1953, it was first described by Irvine and was studied by Gass and Norton in 1966 using fluorescein angiography [1]. As shown in large registry analyses, this complication is still of significant concern despite the many improvements made in intraocular lens technology and surgical methods [2]. It occurs when the blood–aqueous and blood–retinal barriers fail, likely as a consequence of postoperative inflammation, causing fluid to build up in the macula [3,4]. Numerous studies have thoroughly documented the frequency of and risk factors for this illness, underscoring the necessity of efficient preventative and treatment interventions [1,2,3,4,5,6,7,8,9]. IGS has been found to mostly develop 4 to 12 weeks after cataract surgery, manifesting as visual acuity decline and metamorphopsia following early postoperative visual function improvement, with the exception of late-onset IGS [2,3,4,5,6,7,8,9]. In a recent meta-analysis, the incidence rate of clinical IGS was reported to be about 5%, and that of asymptomatic IGS, as detected by fluoresceine angiography, exceeds this proportion [9]. Imaging using optical coherence tomography (OCT) supports earlier and probably more frequent diagnosis of IGS [8]. Advances in cataract surgery with the introduction of small-incision phacoemulsification led to a significant decrease in IGS incidence compared to previously used intra- or extra-capsular cataract extraction [9].

Diabetes mellitus, arterial hypertension, uveitis, and intraoperative complications such as vitreous loss and posterior capsule rupture are among the risk factors that have been described for the development of IGS [10]. A recent meta-analysis confirmed higher incidences in diabetic patients and uveitis but further showed an increased risk in eyes after pars plana vitrectomy or retinal vein occlusion and in eyes with epiretinal membrane [9].

Its pathogenesis, which is believed to be mediated by inflammatory processes induced by surgery, might be exacerbated by the above-mentioned pre-existing conditions that predispose the ocular environment to inflammatory responses [2,3,4,7,9,10].

To reduce the inflammatory response and preserve the integrity of the ocular barriers, anti-inflammatory medications, including corticosteroids and non-steroidal anti-inflammatory medicines (NSAIDs), are used in current treatment regimens [5,10,11]. Previous studies recommend the use of oral acetazolamide, along with corticosteroids and NSAIDs [5,10,11]. Recent studies compared single drugs, combinations, and sequential regimens, as well as systemic and intraocular treatments, showing that personalized therapy based on patient-specific risk profiles may improve resolution rates and reduce recurrence [1,2,9,10,11,12,13,14,15]. To optimize therapy regimens, further research is warranted because the efficacy of various medicines can differ greatly across individuals, which underlines the importance of creating personalized treatment for each patient.

This study aims to compare the treatment regimens used for IGS in clinical practice when administering topical eyedrops alone and/or in combination with oral medication. Through a comprehensive analysis of retrospective data, this study seeks to propose a multidimensional personalized approach to the prevention and management of Irvine–Gass syndrome, enhancing patient outcomes after cataract surgery.

## 2. Materials and Methods

This was a retrospective single-center study that evaluated Irvine–Gass treatment between 2014 and 2023 at Vista Klinik Binningen, Switzerland. This study was approved by the local ethics committee (Ethikkommission Nordwestschweiz EKNZ 2022-00110), performed in accordance with ICH-GCP guidelines, and followed the tenets of the Declaration of Helsinki. According to local requirements, general informed consent for the retrospective analyses of data and the use of imaging material was obtained from the patients.

### 2.1. Subjects

All patients in our electronic database with a diagnosis of postsurgical macular edema following cataract surgery were included. Systemic medical and ocular histories and the following data were evaluated at baseline/follow-up: corrected visual acuity (VA), intraocular pressure (IOP in mmHg, measured with Goldmann applanometry), central subfield thickness (CST in µm, as measured using Heidelberg SD-optical coherence tomography (Spectralis, Heidelberg Engineering, Heidelberg, Germany) in a standard operating procedure (Volume scan 6 mm × 6 mm, at least 19 sections, follow-up mode)), and the time from the start of therapy to the resolution of macular edema (no exudative sub- or intraretinal fluid). For analysis, we compared patients who received local treatment (LT) alone with those who received (additionally or alone) systemic treatment (ST) and patients with a history of cardiovascular diseases (CV) with those without cardiovascular diseases (NCV).

Patients diagnosed with PCME due to Irvine–Gass syndrome with a history of uncomplicated cataract surgery were eligible for this study. If a history of additional surgeries (e.g., pars plana vitrectomy) was found, patients were only eligible when cataract surgery was the last surgery performed. Patients were excluded if they developed IGS following different procedures (e.g., pars plana vitrectomy, trabeculectomy, cornea surgery), had macular intraretinal fluid for other reasons (e.g., uveitis, diabetic macula edema, retinal vein occlusion), or had insufficient imaging material, meaning no sufficient OCT follow-up data since the initial diagnosis.

The diagnosis of PCME due to Irvine–Gass syndrome was made based on SD-OCT (spectral-domain optical coherence tomography) findings of cystoid macular edema and a history of cataract surgery as a standard operation procedure. If necessary, manual correction of OCT layer segmentation was carried out. Visual acuity (VA) was assessed as corrected Snellen VA using an NIDEK auto-refractometer and converted into ETDRS letters [16]. Intraocular pressure was measured using Goldmann applanation tonometry.

### 2.2. Treatment and Procedure

Therapy efficiency was measured based on the remission of macular edema through OCT scans defined as the absence of intra- and subretinal fluid. The time from diagnosis to resolution was measured from the initiation of therapy. For each patient, OCT scans and clinical examination results on the day of diagnosis and following personalized treatment initiation at 2, 4, and 8 weeks; 3 and 6 months; and 1 year were evaluated. When recurrence occurred, no changes were made to the observation periods. Measurements continued to be taken at the previously scheduled time points. For graphical representation, we converted all time points into weeks to ensure uniform time parameters. The 6- and 12-month follow-ups took place after 6 ± 1 months and 12 ± 1 months.

### 2.3. Statistical Analysis

In this study, evaluations were conducted using Python (version 3.10.14, release date 6 December 2021, python.org) as the primary programming language. In the line plots, the markers indicate the calculated estimators (mean) for each group at specific time points. The shaded regions around these markers represent the estimated error intervals: the 50% percentile interval (pi) highlights the central range from the 25th to 75th percentile, while the standard deviation interval (Sd) illustrates the range from [mean − sd, mean + sd], providing insights into the data’s spread around the mean. *p* < 0.05 was defined as significant.

## 3. Results

In total, 136 eyes were followed for a mean of 9.7 ± 15.2 months. Among these 136 eyes, 78 were male (57%), and 58 were female (43%). The average age of the patients was 72 ± 8.5 years. Of the 136 patients, 36 had cardiovascular risk factors, such as arterial hypertension and/or diabetes (26%), and 24 had undergone femto laser-assisted cataract surgery (18%). The phacoemulsification time (*n* = 62) was on average 2.9 ± 2.4 s. IGS occurred in 68% of the patients (*n* = 93) 14 weeks after surgery or earlier. Additionally, 15% (*n* = 21) underwent vitrectomy before cataract surgery, but not as a combined procedure. On average, the time from the initial diagnosis to the full resolution of the edema was 14 weeks ± 14.5 for all patients. Moreover, edema recurred in 21% of the patients (*n* = 28) after the first resolution. All baseline characteristics can be seen in Table 1.

### 3.1. Treatment

**Figure 1:**
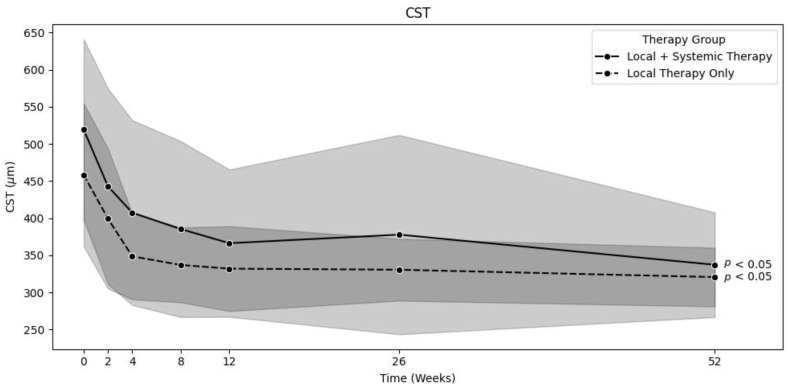
Comparison of decrease in CST over time between locally and systemically treated groups (0 *n* = 75/61, 2 *n* = 75/61, 4 *n* = 72/59, 8 *n* = 58/55, 12 *n* = 46/46, 26 *n* = 36/35, 52 *n* = 31/34). Background color indicates the standard deviation (light grey for combined local & systemic therapy, dark grey for local therapy only).

**Figure 2:**
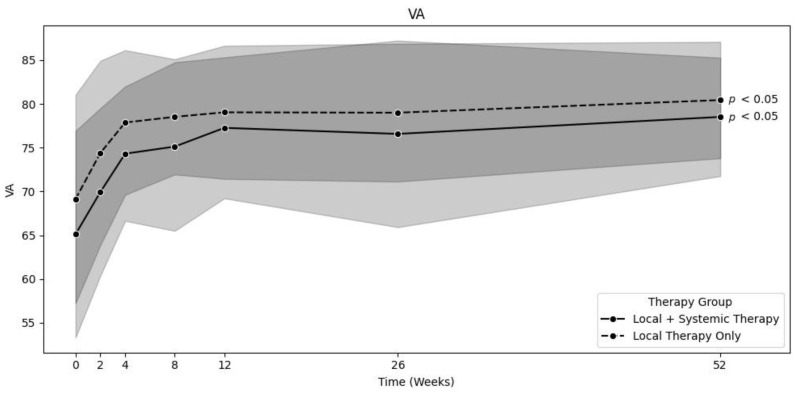
Comparison of increase in VA over time between locally and systemically treated groups (0 *n* = 75/61, 2 *n* = 75/61, 4 *n* = 74/57, 8 *n* = 57/55, 12 *n* = 46/45, 26 *n* = 34/38, 52 *n* = 27/27). Background color indicates the standard deviation (light grey for combined local & systemic therapy, dark grey for local therapy only).

In the locally treated group (LT, *n* = 75), 63 patients were treated with a combination of topical corticosteroids, NSAR, and acetazolamide; 8 patients were treated with topical corticosteroids and NSAR; and 3 patients were treated with only topical corticosteroids. In this group, the mean CST significantly decreased from baseline to 12 months [458.3 ± 96.5 µm to 320 ± 39.5 µm (*p* < 0.01)] [Figure 1]. The mean corrected VA significantly increased from baseline to 12 months [69.1 ± 11.9 to 80.4 ± 6.6 letters (*p* < 0.01)] [Figure 2]. The mean time until the complete resolution of intra- and subretinal fluid (*n* = 74) was 9.5 ± 13.2 weeks, which was significantly shorter than that in the systemically treated group (*p* < 0.05). One patient was lost to follow-up after 6 months, without full resolution of the edema at the last recorded visit. Intraocular pressure remained stable throughout the observation period. Only two patients who were treated with topical corticosteroids had a steroid response, and, as a result, their use was stopped.

The systemically treated group (ST, *n* = 61) comprised patients who received either systemic treatment alone or both topical and systemic treatment from the date of diagnosis. A total of 35 patients received oral corticosteroids, and another 35 received oral acetazolamide inhibitors. The oral corticosteroid and acetazolamide dosages varied and were selected based on each individual patient. Generally, 50 mg of oral corticosteroids and 250 mg of oral acetazolamide (one to three times a day) were prescribed. These dosages were then either reduced or increased depending on the IGS regression rate. Some patients also received multiple systemic treatments. Additionally, although not systemic treatments, 11 of the systemically treated patients received a dexamethasone implant (Ozurdex®, Allergan, an AbbVie company, North Chicago, IL, USA), 3 patients received intravitreal triamcinolone (Kenacort®, Dermapharm AG, Hünenberg, Switzerland), and 1 patient received intravitreal ranibizumab (Lucentis®, Novartis, Basel Switzerland). The mean CST significantly decreased from baseline to 12 months [519.3 ± 121.6 µm to 337.2 ± 70.6 µm (*p* < 0.01)] [Figure 1]. The mean VA significantly increased from baseline to 12 months [65.1 ± 11.8 to 78.5 ± 6.8 letters (*p* < 0.01)] [Figure 2]. The mean time until the complete resolution of intra- and subretinal fluid (*n* = 60) was 19.1 ± 26.3 weeks in the ST group. Here, one patient still had some intraretinal cysts after one year. IOP was also mostly stable over time, with 13 cases of steroid response. Steroid response was defined as IOP > 21 mmHg. The increase in IOD was mostly due to the dexamethasone implant and was treated by administering systemic acetazolamide, withdrawing steroids, or prescribing glaucoma medication such as carbonic anhydrase inhibitors.

The decision as to whether the patients were treated locally or systemically was only made based on the physician’s individual preferences and was administered in a personalized approach for each patient. Baseline differences (e.g., CST, age, sex, and surgery type) did not lead to any therapy adjustments.

### 3.2. Major Edema

**Figure 3:**
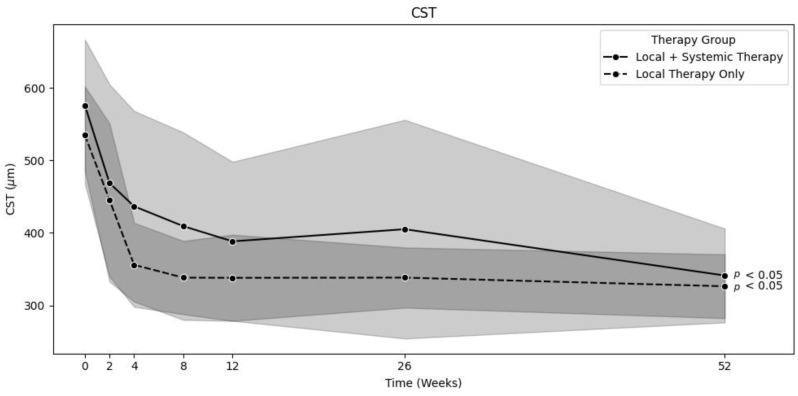
Comparison of decrease in CST over time between locally and systemically treated groups with major edema (0 *n* = 39/44, 2 *n* = 39/44, 4 *n* = 37/43, 8 *n* = 32/40, 12 *n* = 24/32, 26 *n* = 20/24, 52 *n* = 17/23). Background color indicates the standard deviation (light grey for combined local & systemic therapy, dark grey for local therapy only).

To compare the efficacy of local therapy alone and systemic treatment on major edema, we filtered out patients with minor edema to balance the groups. Therefore, we set the minimum baseline size of major macular edema to 450 µm. As a result, there were 38 patients in the LT group, and 43 patients in the ST group. The mean CST decreased from 534.7 ± 67 to 326.4 ± 44 µm (*p* < 0.01) in the LT group and from 575.8 ± 90.8 to 341.3 ± 64.7 µm (*p* < 0.01) in the ST group [Figure 3]. The mean VA increased from 64.2 ± 12.7 at baseline to 79.3 ± 7.6 letters (*p* < 0.01) after one year in the LT group and from 62.5 ± 11.7 to 76.8 ± 7.7 letters (*p* < 0.01) in the ST group. There was no significant difference between these groups, even after filtering out patients with minor edema: a *p*-value of 0.76 was found for the decrease in CST, and *p* = 0.34 was found for the increase in VA.

### 3.3. Risk Factors

**Figure 4:**
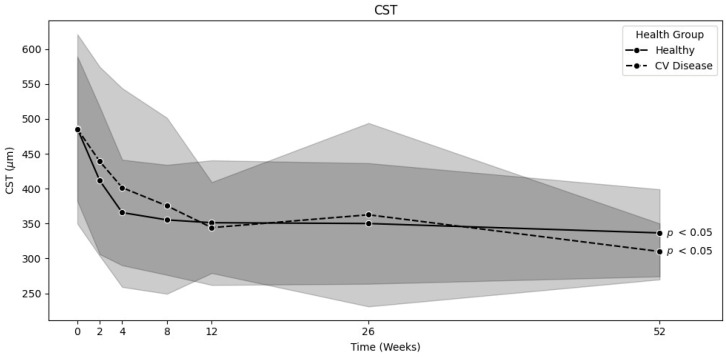
Comparison of decrease in CST over time between the healthy and CV disease groups (0 *n* = 100/36, 2 *n* = 100/36, 4 *n* = 97/34, 8 *n* = 84/29, 12 *n* = 64/28, 26 *n* = 50/21, 52 *n* = 47/18). Background color indicates the standard deviation (light grey for group with CV disease, dark grey for healthy group).

For the healthy and cardiovascular disease (CV) groups, similar results were observed. The mean CST decreased from 485.7 ± 103.4 µm to 336.6 ± 62.4 µm (*p* < 0.01) in the healthy group (*n* = 100) and from 485.4 ± 135.6 µm to 310 ± 40.1 µm (*p* < 0.01) in the CV group (*n* = 36) [Figure 4]. The mean corrected VA increased from 66.3 ± 12.5 to 79.4 ± 6.5 letters (*p* < 0.01) in the healthy group and from 70.1 ± 10 to 79.8 ± 7.5 letters (*p* < 0.01) in the CV group. There was no significant difference between these two groups after one year (CST *p* = 0.09; VA *p* = 0.72). IOP was also stable throughout the entire study period. The time until dry was not significantly different between these two groups, with 13.5 ± 20.7 weeks for the healthy group and 14.7 ± 20.5 weeks for the CV group (*p* = 0.53).

### 3.4. Safety

Overall, the treatment of Irvine–Gass syndrome had only minor side effects. In total, 15 patients experienced a steroid response, which was defined as an IOP higher than 21 mmHg. Most cases were caused by the dexamethasone implant, but it also occurred in two patients who were only treated with local steroids. Other side effects of the systemic treatments such as oral acetazolamide were nausea, dizziness, and paresthesia, noted in only six patients. However, this number could be higher because, usually, not all side effects are recorded in detail in retrospective studies.

### 3.5. Limitations

As this was a retrospective, real-world study, its limitations are mainly loss to follow-up, missing records of therapy side effects, and the population count. Follow-up compliance varied significantly among the patients. Those who were already attending the clinic before therapy (e.g., for regular annual check-ups) demonstrated higher compliance than those who visited only for disease-specific treatment (e.g., referrals from private ophthalmologists). Missing data were not excluded in advance. Instead, all available data at each time point were included in the statistical analysis when given at least a 2-week follow-up. The total sample size (n) was adjusted accordingly for each time point based on the data actually available.

## 4. Discussion

There are no standardized guidelines for the treatment of Irvine–Gass syndrome or postsurgical macular edema since there are many different approaches that can provide a personalized treatment. Most studies suggest initial treatment with topical NSAIDs and corticosteroids [3,4,5,11,12,13,14,15,16]. In a review by Wielders et al., the efficacy of topical corticosteroids and NSAIDs in patients with IGS was shown, which aligns with the findings of our study [17]. A study by Pepple et al. [18] showed the benefits of using oral acetazolamide in addition to topical NSAIDs and corticosteroids, achieving complete and faster resolution of IGS. A recent case report about an off-label use of an intravitreal acetazolamide implant for IGS found an improvement in the edema which was refractory to previous treatments [19]. In the future, this could be a further treatment option, especially as the side effects of oral acetazolamide limit its use as found in a few patients in our study who complained about nausea, dizziness, or paresthesia. However, its use as an additional treatment can be considered when IGS does not improve with topical therapy alone [18]. Further studies evaluated the use of acetazolamide as first-line therapy in patients with IGS, and they found evidence of its efficacy; however, in our study, acetazolamide provided no direct benefits when used as first-line therapy [6,10]. Sane et al. showed that a topical three-drug combination with a corticosteroid, carbonic anhydrase inhibitors, and an NSAID was effective for the treatment of IGS [13].

Targeting the inflammatory pathway as well as reducing vessel leakage favors the use of steroids. Several studies evaluated in recent meta-analyses or systematic reviews, as well as ours, have shown their high efficacy as a local and systemic treatment option [9,10,20,21]. The main concerns about this are an increase in intraocular pressure (steroid response) and, if given as oral medication, systemic side effects [9,10,20,21]. In our study, 15/136 cases developed an IOP increase, which is in line with the current knowledge. Despite not having reported any systemic side effects of oral steroids in our study, we highly recommend involving the patients’ GP in such treatments, especially for applications in higher doses or over a longer term and in diabetic patients [22]. For eyes with limited response to local eyedrops, surface problems due to the drying effect of steroids or in patients who are not able to administer local therapies themselves, intravitreal steroid application might be an option with fewer systemic side effects than oral steroid therapy [23,24,25,26]. While steroid implants provide longer-term effects, the very cost-efficient off-label use of filtered triamcinolone acetate, for example prepared according to the method of Bitter et al. [27], serves as a treatment option for a shorter time period. Probably due to the very good efficacy of the applied therapies and the high proportion of patients treated with systemic steroids in our study, only a few patients received a dexamethasone implant.

While intravitreal application of anti-VEGF substances is the gold standard for the treatment of macular edema due to different causes than IGS, there are only a few reports about successful use in IGS [23,28]. The lack of anti-inflammatory potential of these drugs might be a reason for an unlikely success of anti-VEGF treatment in many IGS cases. But in the future, new immunomodulatory therapies could offer new options [29,30,31], especially if it is possible to apply them locally.

However, it remains unclear which IGS treatment is the most effective. This reinforces the findings of the present study, indicating that topical therapy is non-inferior to systemic therapy. Therefore, every patient can be treated with a personalized approach. Although the current evidence base offers multiple effective treatment modalities, standardized protocols for initiating and escalating therapy in cases of insufficient response are lacking. Future research should focus on well-designed, large-scale randomized controlled trials comparing different topical and systemic treatment regimens, both as monotherapy and in combination. Such studies should also investigate the optimal timing for escalation from topical to systemic therapy, evaluate long-term visual and anatomical outcomes, and assess the safety profile of various agents across patient subgroups. Such evidence will be pivotal in developing standardized, evidence-based treatment algorithms that ensure consistency of care while allowing for individualized patient management. Known risk factors such as diabetes, a history of uveitis, previous retinal vein occlusion, and arterial hypertension are often described in previous studies [3,4,6,7,9,10]. In this study, we compared patients with arterial hypertension and/or diabetes to patients without these conditions and found no difference in therapeutic outcomes. When examining the number of patients with cardiovascular risk factors, it was found that there were fewer patients with such risk factors, but our population is too small to determine whether diabetes and arterial hypertension lead to a higher susceptibility to IGS.

In summary, no significant differences regarding the remission rate of IGS were found in our study. The time until dry was longer in the systemically treated group. This finding shows that some IGS cases are harder to treat than others and thus require systemic or intravitreal treatment (e.g., a dexamethasone implant) as part of a personalized approach. Nevertheless, it seems that, in most cases, patients treated with only topical medication showed a good healing process.

Therefore, it can be postulated that systemic treatment for Irvine–Gass syndrome should be critically assessed in terms of possible side effects and affected organs such as the kidney, for example, when administering acetazolamide to elderly patients. As the same results were obtained when treating major edema, it can be assumed that local treatment being on par with systemic treatment is not a coincidence.

## 5. Conclusions

There are currently no standardized guidelines for the treatment of Irvine–Gass syndrome. However, based on the findings of this study, it appears reasonable to initiate management with topical therapy alone, personalized for each individual patient. If there is no sign of regression in IGS after a defined period, systemic treatment should then be considered. As we only examined local therapy in general, no conclusions can be drawn regarding the optimal choice of initial topical treatment.

The results of our study suggest that, in cases of IGS in patients with cardiovascular risk factors such as arterial hypertension or diabetes, treatment does not necessarily need to be adjusted compared to that of otherwise healthy individuals.

## Figures and Tables

**Table 1:** Baseline characteristics of the total population and subgroups.

Baseline Characteristics	
Total population (*n*)	136
Females; %	58; 43%
Males; %	78; 57%
Age (years ± SD)	72 ± 8.5 years
Follow-up time (months ± SD)	9.7 ± 15.2 months
Cardiovascular risk factors (n; %)	36; 26.5%
Healthy (*n*, %)	100; 73.5%
Femto laser-assisted surgery (n; %)	24; 17.6%
Phakotime in seconds (*n* = 62; ±SD)	2.9 ± 2.4 s
Following vitrectomy (*n*; %)	21; 15.4%
Baseline CST in LT ±SD	458.3 ± 96.5 µm
Baseline CST in ST ± SD	519.3 ± 121.6 µm
Baseline visual acuity in LT ± SD	69.1 ± 11.9 letters
Baseline visual acuity in ST ± SD	65.1 ± 11.8 letters
Baseline CST in CV ± SD	485.4 ± 135.6 µm
Baseline CST in NCV ± SD	485.7 ± 103.4 µm
Baseline visual acuity CV ± SD	70.1 ± 10 letters
Baseline visual acuity NCV ± SD	66.3 ± 12.5 letters

SD, standard deviation; CST, central subfield thickness; LT, locally treated group; ST, systemically treated group; CV, cardiovascular group; NCV, healthy group.

## Data Availability

The raw data supporting the conclusions of this study will be made available by the authors, without undue reservation and under consideration of ethical restrictions, after request to EKNZ.
